# Rustims: An Open-Source Framework for Rapid Development and Processing of timsTOF Data-Dependent Acquisition Data

**DOI:** 10.1021/acs.jproteome.4c00966

**Published:** 2025-04-22

**Authors:** David Teschner, David Gomez-Zepeda, Mateusz K. Łącki, Thomas Kemmer, Anne Busch, Stefan Tenzer, Andreas Hildebrandt

**Affiliations:** † Institute of Computer Science, Johannes-Gutenberg University, 55128 Mainz, Germany; ‡ Institute for Quantitative and Computer Biosciences (IQCB), Johannes-Gutenberg University, 55128 Mainz, Germany; ¶ Helmholtz Institute for Translational Oncology (HI-TRON) Mainz - a Helmholtz Institute of the DKFZ, 55131 Mainz, Germany; § German Cancer Research Center, DKFZ, 69120 Heidelberg, Germany; ∥ University Medical Center, Johannes-Gutenberg University, 55131 Mainz, Germany

**Keywords:** mass spectrometry, proteomics, ion mobility, timsTOF, DDA-PASEF, framework, rust-lang, Python, open-source

## Abstract

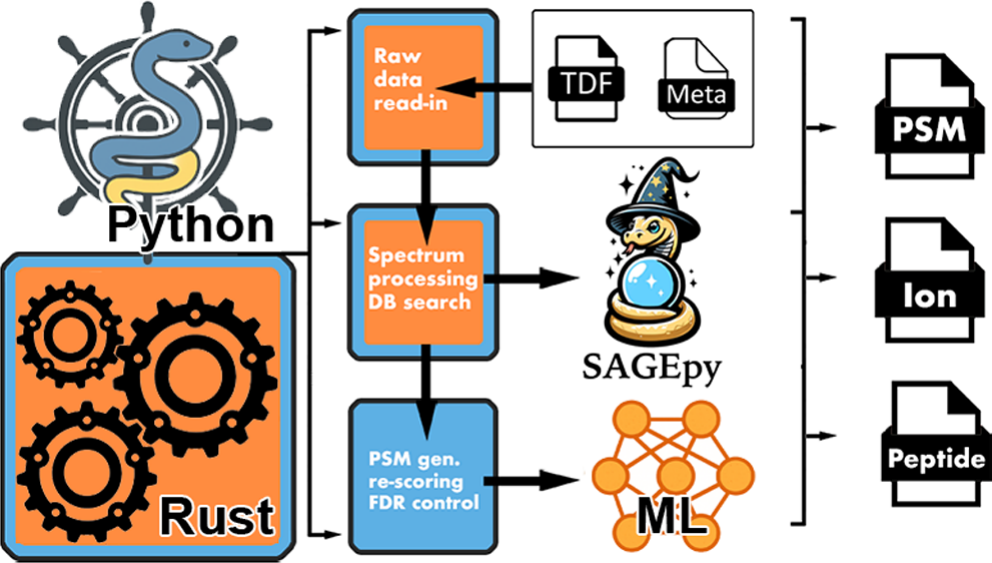

Mass spectrometry is essential for analyzing and quantifying biological samples. The timsTOF platform is a prominent commercial tool for this purpose, particularly in bottom-up acquisition scenarios. The additional ion mobility dimension requires more complex data processing, yet most current software solutions for timsTOF raw data are proprietary or closed-source, limiting integration into custom workflows. We introduce rustims, a framework implementing a flexible toolbox designed for processing timsTOF raw data, currently focusing on data-dependent acquisition (DDA-PASEF). The framework employs a dual-language approach, combining efficient, multithreaded Rust code with an easy-to-use Python interface. This allows for implementations that are fast, intuitive, and easy to integrate. With imspy as its main Python scripting interface and sagepy for Sage search engine bindings, rustims enables fast, integrable, and intuitive processing. We demonstrate its capabilities with a pipeline for DDA-PASEF data including rescoring and integration of third-party tools like the Prosit intensity predictor and an extended ion mobility model. This pipeline supports tryptic proteomics and nontryptic immunopeptidomics data, with benchmark comparisons to FragPipe and PEAKS. Rustims is available on GitHub under the MIT license, with installation packages for multiple platforms on PyPi and all analysis scripts accessible via Zenodo.

## Introduction

Mass spectrometry (MS) is a key technology for the elucidation of chemical composition and quantification of biological samples. It is used to determine the mass-to-charge ratio (*m*/*z*) of ionized molecules with high sensitivity and precision and is being implemented in an expanding variety of applications. For proteomics, bottom-up strategies are widely used for exploratory studies, where as many peptides and proteins as possible present in a given sample should be detected and quantified. There, proteins are digested into smaller peptides, often using enzymes that make deterministic cuts after known motifs of amino acids. One key application is to study protein dynamics regulated by biological conditions, e.g., whose levels differentiate between ill and healthy individuals. To achieve this, various data acquisition strategies have been set in place, with more prominent examples being Data Dependent Acquisition (DDA) and Data Independent Acquisition (DIA).^1,2^ Finding the optimal data acquisition strategy for a specific research question remains an object of a very active research.^3^ In this context, the Bruker timsTOF is a widely adopted commercially available platform, typically paired with liquid chromatography separation (LC). The timsTOF combines trapped ion mobility separation (TIMS) and *m*/*z* measurement using a fast scanning time-of-flight mass analyzer (TOF), allowing for fast analyses and deep proteome coverage.^4^ This is facilitated by the parallel accumulation-serial fragmentation acquisition strategy (PASEF) that takes advantage of the correlation between the mass-to-charge ratio and ion mobility of the peptide ions. While the timsTOF provides exciting new acquisition layouts, the resulting raw-data is of increased complexity both in terms of recorded dimensions and the total number of events recorded. Moreover, collected signals possess a lot of additional structure that need to be taken into account when processing such data. When it comes to available processing software, one is faced with a variety of options to choose from. However, currently existing open-source software solutions often lack in support for ion mobility measurements. Complete solution tools taking into account the specifics of the raw data are mostly commercial, such as PEAKS^5^ or Spectronaut,^6^ or made available without fully disclosing the code base, like MSFragger,^7^ MaxQuant,^8^ or DIA-NN.^9^ We therefore see a need to provide tools designed to take into account the specifics of the timsTOF raw data but at the same time are open, free, and can be integrated into existing workflows and pipelines. Just recently, there have been new projects released that also picked up this idea,^10−12^ demonstrating its importance (detailed comparisons in section Software Requirements and Existing Open Source Tools).

We present here rustims, a framework composed of Rust and Python packages that allows to quickly build solutions for timsTOF raw data processing. rustims is designed as a modular, reusable collection of components. We demonstrate its capabilities by building a pipeline to process DDA-PASEF raw data including rescoring. Then, by integrating a recently published release of the Prosit intensity predictor^13^ optimized for timsTOF data, we show that our software interoperates well with existing free-and-open-source solutions built by others.

The outputs from our pipeline are fully compatible with the recently introduced TIMS^2^Rescore,^12^ a rescoring tool specifically tailored for timsTOF data. This integration makes our framework particularly well-suited for immunopeptidomics raw data processing, where practical open-source solutions are still scarce.

Besides, we extended the ionmob model for ion mobility predictor, including additional post-translational modifications (PTMs) measured in a recent study.^14^ We exemplify the usage of the pipeline including rescoring for tryptic proteomics and nontryptic immunopeptidomics DDA-PASEF data, and compare the results with outputs from FragPipe and PEAKS. rustims is a valuable addition to the free software domain, enabling quick prototyping and fine-grained, customizable processing of raw timsTOF DDA data.

### Software Requirements

Processing of (timsTOF) raw DDA data can be broadly divided into four phases. The first phase involves reading raw data from disk, which gets stored during acquisition in a vendor-proprietary format. This step typically requires either converting the data into an intermediate open format, such as mzML, or using tools capable of directly interacting with vendor read-out binaries. In the second phase, loaded raw precursor and fragment spectra are translated into lists of peptide and protein identifications. It often includes extracting quantitative information for peptides and proteins and is usually the most computationally intensive step, as it involves peak and feature detection as well as signal integration for quantification and database construction and searching for identification. This is especially true for timsTOF data, since the additional ion mobility dimension greatly increases data set complexity compared to traditional LC-MS/MS data sets, with experiments potentially generating millions of spectra and billions of peaks. The third phase requires statistical analysis to provide confidence estimates for the success of identification and quantification. In recent years, a fourth phase has gained popularity: the rescoring postprocessing using machine learning techniques to increase the number of confidently identified candidates.

To process the data efficiently, high performance and parallelism are needed, including the potential use of dedicated hardware. This makes a compiled, strongly typed language preferable, as it tends to result in more robust, testable, and collaboratively developed software. However, this language preference might make the framework harder to integrate into existing workflows, often implemented in a scripting language like Python. Additionally, postprocessing software designed to boost identifications heavily relies on modern machine learning frameworks, which are also predominantly Python-centric.

### Existing Open Source Tools

The research community has developed a variety of free and open-source tools able to interact with timsTOF data, oftentimes addressing specific steps in the pipeline. OpenTIMS^15^ is a C++ library with bindings for Python, R, and Java, designed for streaming timsTOF raw data into RAM, one or multiple frames at a time. AlphaTims,^16^ a pure Python library, provides similar functionality but focuses on random access to timsTOF data. It achieves this either by loading data fully into RAM or by converting the vendor format to a custom HDF-based format first. The timsrust project,^17^ implemented entirely in Rust, enables data read-out without relying on vendor binaries. However, this approach is currently only suitable in scenarios where potentially high deviations from vendor-provided functions for translating raw index values into physical values are acceptable (see subsection rustdf in section Approach and Software Architecture).

I2MassChroq^11^ provides an identification and quantification workflow written in C++ and uses X!Tandem^18^ for peptide identification. Similarly, AlphaPept^10^ is a Python-based collection of tools that leverage the Alpha ecosystem. To enhance performance for computationally intensive tasks, AlphaPept relies on just-in-time (JIT) compilation using Numba^19^ and employs additional vendor binaries for feature detection with timsTOF data.

Postprocessing tools such as Oktoberfest,^20^ AlphaPeptDeep,^21^ and MS^2^Rescore^22^ specifically handle timsTOF-specific features, including predictions for the additional ion-mobility dimension and PASEF fragmentation patterns. These tools are implemented in Python, leveraging modern machine learning libraries like TensorFlow,^23^ PyTorch,^24^ and scikit-learn.^25^

## Approach and Software Architecture

While it is possible to create comprehensive workflows using the tools described above, such as the implementation by AlphaPept,^10^ we aimed to provide a toolbox that focuses on three core principles: (1) a strong focus on timsTOF data, making platform-specific features accessible; (2) ease of use and hackability to support rapid prototyping of new ideas; and (3) a library core implemented an efficient, general, type-safe, and memory-safe language. This section describes our approach to meet our software requirements and introduces several new libraries we have developed for this purpose.

rustims employs a two-language approach, similar to OpenMS.^26^ Implementations of algorithms and processing steps that demand high performance and parallelism are provided in a strongly typed, lower-level language, Rust. While Rust is not yet as widely adopted as C++ for low-level scientific computing tasks, it is increasingly recognized within the scientific community for its modern design, ease of parallelization, and simplified build process.^27^ This growing popularity has reached the mass spectrometry community, as shown by the recent and actively maintained rusteomics initiative,^28^ which develops free and open-source solutions specifically for mass spectrometry data analysis.

**Figure 1:**
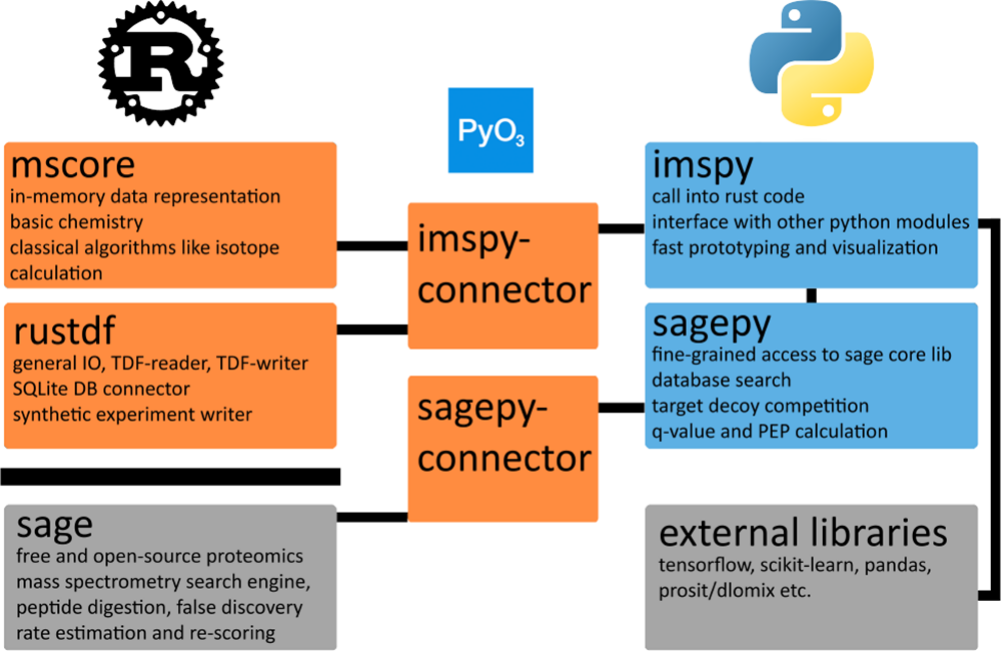
Software architecture of rustims. The architecture includes two Rust-only libraries, mscore and rustdf, which are interfaced with Python through the imspy-connector, a Rust library utilizing PyO3. The connector is wrapped by our pure Python library, imspy, which further integrates with other Python libraries such as TensorFlow for machine and deep learning, as well as proteomics-specific tools like Prosit. imspy also incorporates the Python package sagepy together with sagepy-connector, our binding to the Sage search engine to generate initial PSMs and perform false discovery rate control via target decoy competition.

Our created tools and algorithms are exposed to the end-user through a scripting language, here Python, as visualized in Figure 1. This design makes it straightforward to interface with existing Python libraries and machine learning models provided by others. The core difference compared to OpenMS is a clear focus of our framework on timsTOF data. This makes the code base focused on providing building blocks that are explicitly tailored to fit the complex acquisition layouts of this platform.

### rustdf

Bruker stores data generated by timsTOF devices in a custom format called TDF. This format includes an SQLite database (analysis.tdf) that contains metadata about the experiment, acquired frames, and, depending on the PASEF acquisition method used, information about targeted precursor ions (DDA) or wider quadrupole selection windows (DIA). Raw peaks (analysis.tdf_bin) are stored as a compressed binary block of indexed values for scan, TOF, and intensity, using a custom compression scheme. rustdf implements a custom reader written in Rust for the data set but relies on Bruker proprietary binaries to translate the indexed values of scan and TOF to inverse ion mobility and *m*/*z*. This is necessary since currently the concrete implementations of these translation functions are not disclosed. We plan to include open calibrated translation functions, such as those available in I2MassChroq, in a future release of the software. These Bruker binaries are called from Rust via the Foreign Function Interface (FFI) and for simplicity are provided on PyPi.^29^ This dependency on the Bruker binaries unfortunately renders integrating with MacOS operating systems so far impossible since Bruker does not provide binaries for that platform, at least not without use of emulation/virtualization methods, such as those offered by Docker.^30^

The rustdf crate provides functionality to establish a connection to serialized timsTOF raw data on disk, handling decompression and translation of indexed values. The smallest loadable unit is a single frame, which is given by the compression scheme implemented by Bruker. When called via the Python binding, the reader has functionality comparable to other readers presented before, with a few differences: unlike alphatims,^16^ which loads the raw data once and optionally translates it to a custom hdf5 based format, rustdf is designed for streaming raw data into random access memory directly from the vendor format. Compared to OpenTIMS,^15^ it offers an object-oriented layout for spectra, frames, and slices, which is beneficial for software development focused on raw data processing. It is also possible to return the data as pandas data frames or numpy arrays, if the user so chooses.

### mscore

The mscore crate provides in-memory representations for timsTOF data. The core data structure is a TimsFrame, one retention time point holding a collection of scans where scans represent mass spectra that are separated by their ion mobility. TimsFrames can be either decomposed into their individual TimsSpectra, or collected into TimsSlices that represent some number of (not necessarily) consecutive frames, e.g., a full cycle of the timsTOF experiment. These data structures provide convenience methods for filtering, binning, vectorization, and some mathematical operators to add-up multiple objects of type TimsFrame, multiply them with a scalar etc. Furthermore, the mscore crate also provides basic chemistry functionality allowing to calculate expected masses and isotope distributions for precursor and fragment ions of peptides in silico.

### sagepy

The Sage project^31^ consists of a collection of Rust crates that implement a proteomics search engine to generate peptide spectrum matches (PSMs), perform false discovery rate (FDR) estimation, rescoring, and label-free quantification (LFQ). By implementing the fragment-based indexing strategy of the reference database first proposed by MSFragger,^7^ Sage efficiently utilizes multicore systems and achieves very high speed in scoring candidate spectra, running an order of magnitude faster than the already rapid MSFragger tool.^31^ Available as a command-line tool, Sage is developed as free and open-source software, accessible on GitHub.^32^

We developed a Python binding to the Sage core library, sagepy-connector, using the PyO3 library,^33^ which exposes key operations and data structures. These include protein digestion settings, reference database generation, raw-data preprocessing, spectrum scoring, score-ranked retrieval of PSMs, and computed PSM statistics such as retention times, ion mobilities, matched b/y ions, and their intensities. Sage’s internal features for LFQ are also exposed; however, at this time, timsTOF data is unsupported due to the complexities of tracking peaks in the additional inverse ion-mobility dimension. To enable FDR control, we implemented target-decoy competition for *q*-value calculation and PTM filtering, following the methods proposed by Crema,^34^ including PSM-level, peptide-level, and double competition approaches.

The Python package sagepy provides a pythonic wrapper around the binding code and can be easily installed via package managers like pip. The library is not limited to timsTOF data but can be used to search spectra coming from any source, as long as they are passed from Python. This easy-to-use yet extensively configurable interface allows researchers to integrate Sage more flexibly into workflows that utilize libraries for machine and deep learning, such as TensorFlow^23^ or PyTorch.^24^ For example, it allows to acquire new training or fine-tuning data to update the models for tasks such as retention time, ion mobility, or fragment ion intensity prediction (see section Rescoring of PSMs for more details).

### imspy

The imspy library is a Python library integrating our Rust crates and their respective functionality through a binding library called imspy-connector. It is the main interface for users to work with any of the here presented functionality. Besides exposed data access and in-memory representations of timsTOF raw data, imspy also provides several in-house developed deep-learning models for retention time and ion mobility prediction. It also contains the latest version of our ionmob predictor,^35^ extended to additional post-translational modifications (PTMs) measured in a recent study.^14^ The model declarations have been updated to be compatible with the Keras 3^36^ Python library, allowing to run models not only with TensorFlow^23^ but also Pytorch^24^ or JAX,^37^ frameworks routinely utilized for tasks such as rescoring.

## Material and Methods

### Hardware

Development of the pipeline, including model training, package development, and the execution of FragPipe were performed on a workstation running Ubuntu 22.04 with 32 GB of RAM, an AMD Ryzen 7 3700X 8-Core Processor (16 threads) and a NVIDIA GeForce RTX 2070 GPU. The imspy_dda pipeline was executed on the above-described setup as well as for the very large search space encountered during immunopeptidomics data processing on a workstation running Linux (Ubuntu 22.04), equipped with a Ryzen Threadripper 3990X CPU boasting 64 logical cores (128 threads), 256 GB of RAM, and an NVIDIA GeForce RTX 4090 GPU.

### Software

All software was developed using Rust (version 1.77.2), Python (version 3.11), TensorFlow 2.15.1, CUDA version 11.2, and cuDNN 8.1.1. The Rust library PyO3 (version 0.21.2) was utilized to expose Rust functionalities to Python. TIMS^2^Rescore as part of MS^2^Rescore 3.1.4 and Mokapot 0.10.0 were used for rescoring. The software depends on various other libraries; a detailed list of these dependencies can be found in the rustims project repository on GitHub and the Zenodo repository. FragPipe version 22.0 was used for tryptic data sets of HeLa samples, PEAKS version XPro was used for HLA immunopeptidomics data. sagepy was built with Sage version 0.15.0-alpha.

### Deep Learning Model Training, Fine-Tuning, and Rescoring

All in-house developed deep learning models have been trained using the strategy described for the ionmob predictor,^35^ randomly splitting data into training, validation, and test sets. The retention time predictor was pretrained on data available from dlomix resources at GitHub.^38^ The ionmob GRUPredictor for ion mobility prediction was pretrained using the same data sets used previously, but extended by recently published data with additional PTMs.^14^

During the imspy_dda pipeline execution, the retention time and ion mobility predictors were fine-tuned with the following strategy: for both models, PSMs with a *q*-value of 1% and below were collected. The PSMs were then randomly split on peptide level into sets of 80% training and 20% validation, making sure that no peptides were present in training that were used for validation, and the models were fine-tuned. This was done by consecutively lowering the learning rate between 10^–3^ and 10^–6^, measuring model performance gains on the validation data while fitting on the training data, and decreasing the learning rate if model performance on the validation data did not increase for 5 epochs or stopping after the learning rate hit the lower bound.

Rescoring was performed by first randomly splitting all PSMs on peptide level into five nonoverlapping batches. We then performed a leave-one-out prediction strategy, where four out of five batches were used to fit an LDA classifier to optimally separate target from decoy hits, using all decoy hits but only target hits with an initial *q*-value of 1% and below. The trained model was then used to predict the portion of the data that was not in the training set, consequently performing the training and prediction five times.

### Search Engine Settings

For the tryptic data sets, sagepy was configured as follows: the reference proteome was in silico digested using trypsin settings, allowing up to 2 missed cleavages. Carbamidomethylation of cysteine was set as a fixed modification, N-terminal acetylation and methionine oxidation were set as variable modifications. Precursor tolerance was set to ± 15 ppm, fragment tolerance was set to ± 20 ppm, minimum matched peaks was set to 5, minimum fragment *m*/*z* was set to 150 Da, maximum fragment *m*/*z* was set to 1700 Da, maximum precursor charge was 5, maximum fragment charge was set to 2. Top-n peak selection from fragment spectra was set to 150. Minimum peptide length was set to 7, maximum peptide length was set to 30. FragPipe and MSBooster were run with the Default workflow, setting precursor mass tolerance and peptide length the same as sagepy. All results were filtered at an FDR of 1%, removing decoys.

For the HLA immunopeptidomics data sets, search parameters of sagepy were kept the same except precursor tolerance which was set to ± 20 ppm. Digestion and PTM settings were set differently: unspecific cleavage was used for proteome digestion, allowing peptides between 7 and 25 amino acids. No fixed modifications were set, but oxidation of methionine, N-terminal acetylation and cysteinylation were set as variable modifications. Database split was set to 6, randomly assigning proteins into batches and searching them consecutively. The resulting PSMs where then merged. FragPipe and MSBooster were run with the Nonspecific-HLA workflow, but precursor mass tolerance and peptide length where set as done for sagepy. All results were filtered at an FDR of 1%, removing decoys.

### LC-IMS-MS Raw Data

Public data sets were used. The raw data is available in ProteomeXchange or jPOSTrepo with the identifiers mentioned in the text below.

Tryptic proteomics (PXD043026, JPST002158).^35^ Briefly, HeLa whole cell lysates were digested with trypsin by filter-aided sample preparation (FASP) and analyzed in a nanoAcquity LC (Waters) coupled with a timsTOF Pro-2 in DDA-PASEF mode. Peptides were directly injected into a reversed-phase C18 column (Aurora 25 cm x 75 μm 1.6 μm, IonOpticks) and separated by gradients increasing the proportion of mobile phase B (1% formic acid in acetonitrile) to phase B (1% formic acid in water). HeLa digests were analyzed using three different gradient lengths (20, 47, and 110 min) to generate data sets with different complexity (HeLa-20 min, HeLa-47 min, and HeLa-110 min, respectively).

HLA class I immunopeptidomics (HLA1 ps, identifiers: PXD040385, JPST002044).^39^ Briefly, JY (CVCL_0108) HLA1 ps were enriched by immunoprecipitation and injected at 1, 2, 5, 10, 20, or 40 million cells equivalents using Thunder-DDA-PASEF.

### Pipeline for DDA-PASEF Data Processing

**Figure 2:**
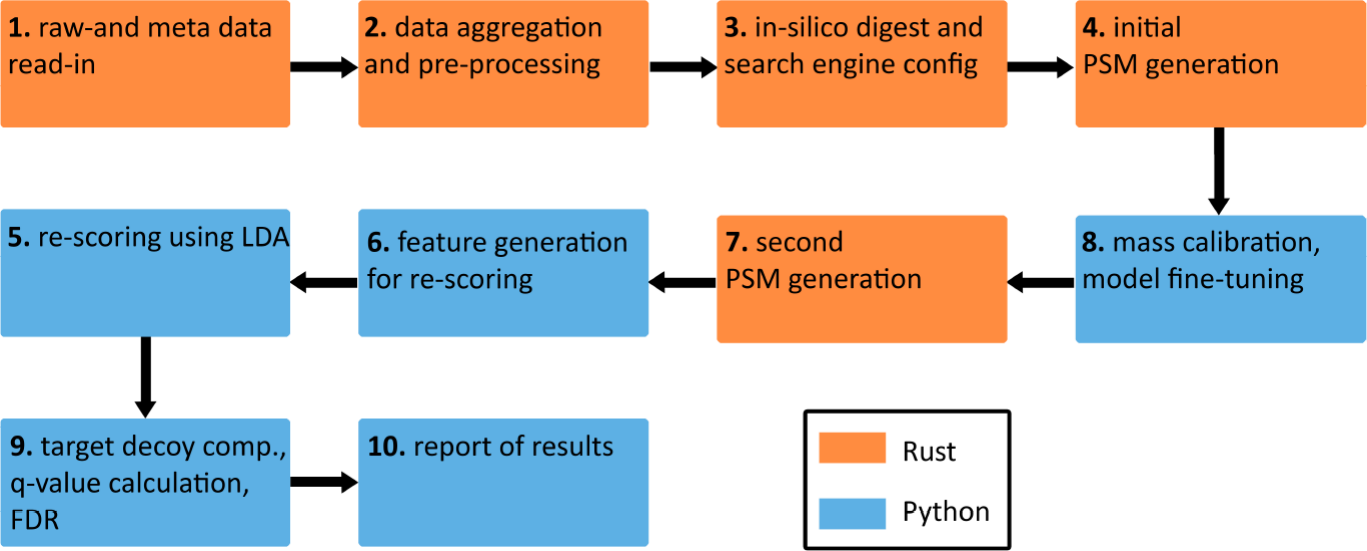
imspy_dda pipeline implemented using the rustims toolbox to process DDA-PASEF data. Pipeline parts implemented in Rust are shown in orange, and Python in blue. It is important to note that all pipeline parts are called from Python (imspy), using bindings generated with PyO3. The pipeline comprises ten consecutive steps, including raw data read-in, refragmented data aggregation and preprocessing, search space generation, scoring configuration, PSM generation, mass calibration, model fine-tuning, rescoring, and finally target-decoy competition for false discovery rate estimation and report of results.

The framework facilitates building and testing of peptide identification workflows. To demonstrate this, we built a processing pipeline, named imspy_dda, for timsTOF DDA-PASEF raw data, see Figure 2. The individual steps and corresponding user functionality are described in detail in the following sections.

#### Raw Data Read-In

Using the Python interface to the rustdf crate, precursor ion information including charge state, monoisotopic peak, quadrupole selection window, and scan dimension window are loaded from the meta data. Fragment spectra are extracted from the raw data and filtered in the scan dimension according to the selection window set by the instrument. They are joined into a mutual table.

#### Data Aggregation and Spectrum Preprocessing

In DDA mode, the timsTOF refragments ions if the fragment spectrum falls below a specific intensity threshold. Precursors targeted multiple times are merged by the pipeline into a single fragment spectrum. These spectra are then transformed into a Sage-compatible format that retains only the top-N peaks in each fragment spectrum.

#### Search Engine Configuration

A Sage reference database is created by passing a FASTA file with the reference sequences, along with settings for an in silico digest and expected static and variable PTMs. Subsequently, a Sage scorer is configured, with parameters such as the number of matches per spectrum to return, ppm error tolerances, and minimum matched peaks.

#### First PSM Generation

The configured scorer searches the database using the processed spectra from the previous step, generating a collection of Peptide Spectrum Matches (PSMs). These PSMs contain features directly available from the Sage search, such as hyperscore, average ppm error per spectrum, and matched fragments.

#### Mass Calibration, CE Calibration, and Model Fine-Tuning

The ppm errors returned for each spectrum are used to compute a global median ppm error at the experiment level and used to correct the searched fragment spectra. The top scoring 4096 PSMs are used to calibrate the collision energies (CE) reported for each fragment spectrum using the Prosit intensity predictor. Additionally, *q*-values are calculated at the PSM level, and PSMs with *q*-values ≤ 0.01 are selected for fine-tuning retention time and ion mobility predictors (as detailed above).

#### Second PSM Generation

The corrected spectra are searched against the reference database again, producing a collection of PSMs for rescoring.

#### Feature Generation for Rescoring

Rescoring is implemented as part of the imspy_dda pipeline. To build up the feature space, all features available from the sagepy database search in addition to the predicted intensities provided by a release of Prosit intensity predictor optimized for timsTOF data, an updated version of our ionmob model and a custom retention time predictor are used. This results in a set of 21 scalar descriptors for each PSM, incorporating all features returned directly by Sage, along with deltas of predicted retention times and ion mobilities and features derived from observed and predicted fragment intensities.

#### Rescoring of PSMs

Linear discriminant analysis (LDA) from scikit-learn^25^ is used as the target-decoy separation engine. It is the same type of classifier implemented by the Sage command line tool.^31^ The model is trained to optimally distinguish between target and decoy hits (detailed above). The distance from the decision boundary is computed, creating a new score for rescoring. Additionally, mokapot^40^ can be used to rescore and control the FDR of generated PSMs, facilitating result comparison with established software.

#### Target Decoy Competition

Target-decoy competition is conducted at both the PSM and peptide level, using a double competition strategy for peptides.^34^

#### Report of Results

All PSMs, along with generated features, are serialized to JSON and stored in binary format. Results at both PSM and peptide levels are filtered at a 1% FDR threshold and saved to disk as.csv files.

## Results and Discussion

### Performance on Tryptic Data

**Figure 3:**
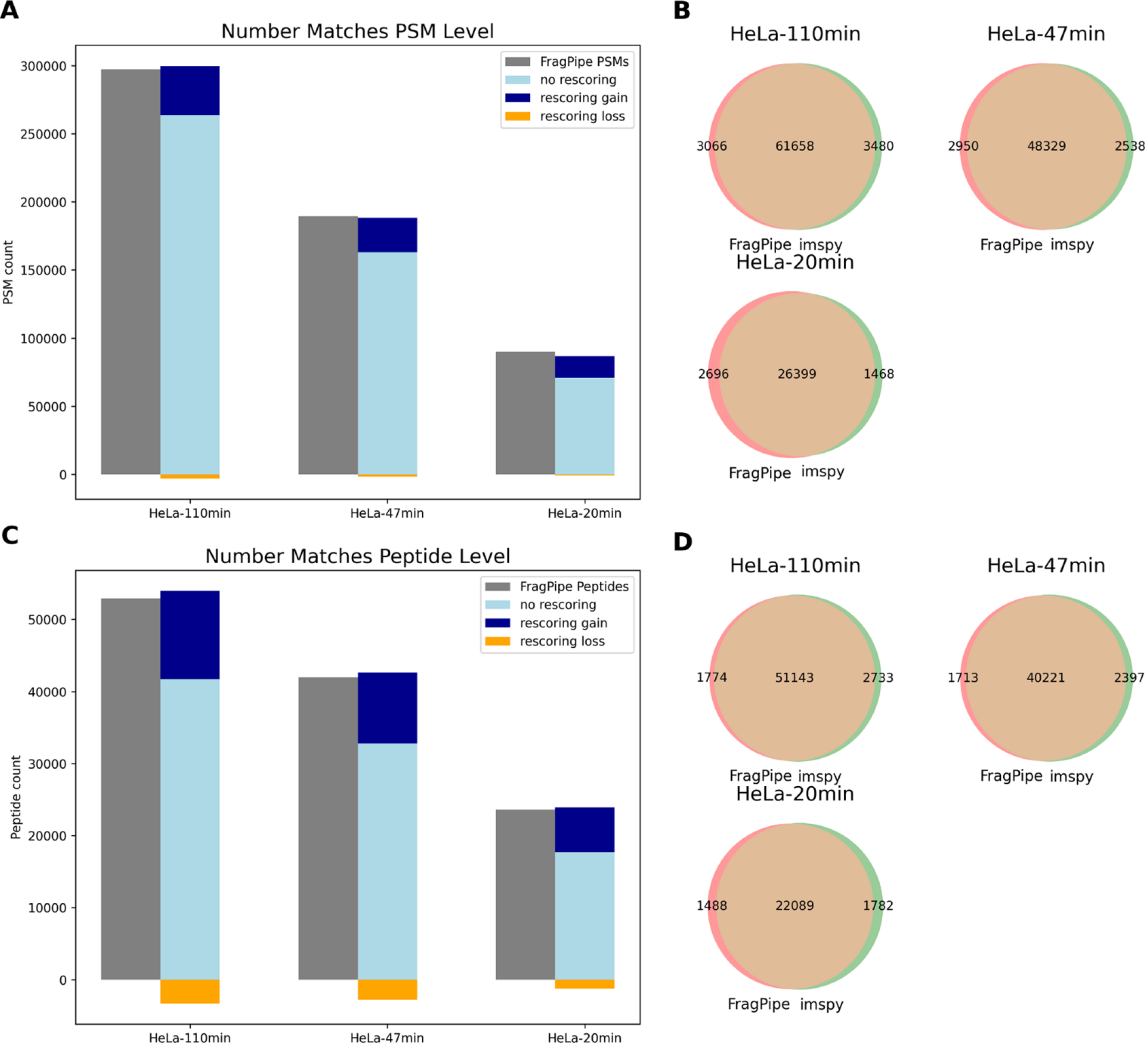
Performance of imspy_dda pipeline vs FragPipe + MSBooster on different gradient lengths of tryptic HeLa digest acquired with DDA-PASEF. (A, C) Total number of identified PSMs (upper) and peptides (lower) at 1% FDR, showing that the identification pipeline presented here is able to achieve good performance on such data compared to state-of-the-art software. (B, D) Overlap of identified ions (upper) and peptides (lower) at 1% FDR, showing high overlap for identifications from both tools.

To exemplify the usage of the pipeline, we processed a collection of tryptic HeLa digests of varying gradient lengths using both our tool and the well-established FragPipe tool. We named these data sets HeLa-20 min, HeLa-47 min, and HeLa-110 min, corresponding to the gradient lengths used for the analysis (20, 47, and 110 min, respectively). Generally, we achieved identification rates comparable to results from FragPipe across all gradient lengths (Figure 3A, C). We also observed high overlaps in identifications at both the ion and peptide levels (Figure 3B, D). Rescoring improved identification rates, with the similarity between predicted and observed ion intensities and predicted retention times having the highest impact on the classification capability, in addition to the initial hyper score. When using mokapot as a postprocessor after the generation of the feature-space, the number of identifications where even slightly higher, see Figure S1), and also slightly higher when using the Tims^2^Rescore rescoring tool, see Figure S4). We still include our own rescoring module since this shows the capabilities of the framework to provide the flexibility to choose between published strategies, or customizing a model for the most fine-grained control.

To test the performance of database query extraction from raw data, we compared results of running pure Sage as a command line tool, using MGF files generated by the Bruker vendor software Compass DataAnalysis and by passing the TDF files to Sage directly. We found that using extracted MGF files as input yielded roughly 5% more significant results compared to our pipeline or using Sage as a command line tool with TDF input (Figure S3). These findings suggest that spectral preprocessing could still be further optimized. To give users the most flexible choice, we also provide an option to run our pipeline using MGF files generated by Compass DataAnalysis instead of raw data sets.

### Performance on HLA Data

**Figure 4:**
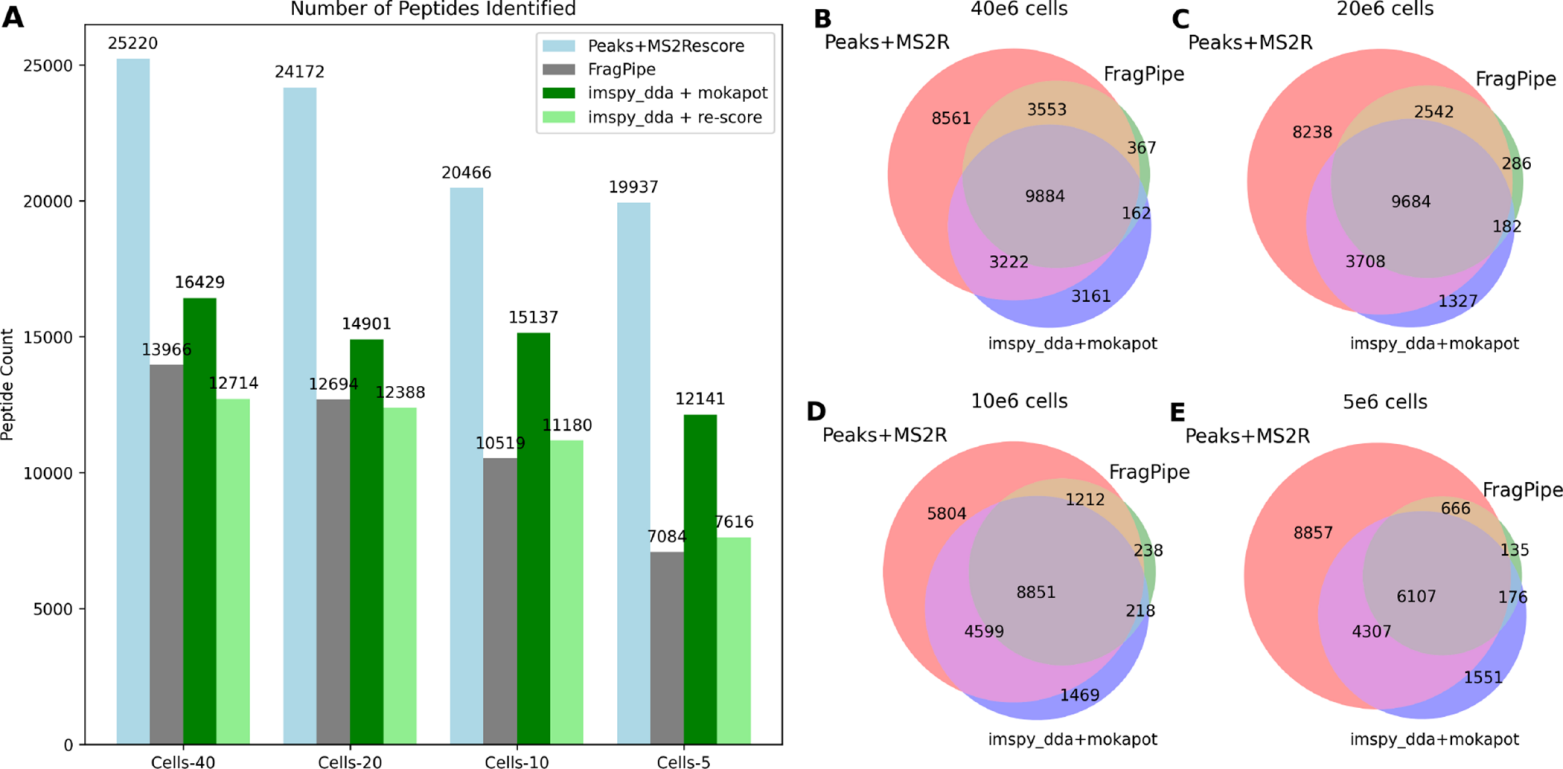
Performance of imspy_dda pipeline with and without mokapot vs Peaks + MS^2^Rescore (v3.0.0b4, timsTOF model) and FragPipe + MSBooster on HLA class I ligand peptides (HLA1p) acquired with Thunder-DDA-PASEF.^39^ (A) The performance is shown for different amounts of cells per sample, ranging from 5 × 10^6^ to 40 × 10^6^ cell equivalents. (B–E) Set comparison of identified peptides between imspy_dda.

To evaluate if our pipeline is also applicable for identifications in more difficult search spaces, we reanalyzed raw data recently published with an immunopeptidomics-tailored acquisition scheme called Thunder-DDA-PASEF.^39^ One of the difficulties encountered with such data is the fact that HLA ligand peptides are not generated by a single specific protease and therefore very large search spaces based on nonspecific cleavage need to be constructed when processing it using an in silico generated reference database. As the original Sage tool was initially not designed for this task, we developed a strategy to process the reference database in chunks, similar to FragPipe. We provide this as a part of our pipeline, where it is now possible to split a given reference proteome fasta file into multiple parts, search the parts sequentially, and merge the results. Results of processing samples of differing cell concentrations of HLA1p peptides are shown in Figure 4A).

We compare them to results published with the Thunder-DDA-PASEF method, where PEAKS was used together with MS^2^Rescore to generate PSMs and boost identifications. Additionally, we added results from reanalyzing the data using FragPipe with MSBooster. Our tool identifies approximately 60% as many peptides as PEAKS with MS^2^Rescore but yields more identifications at lower sample concentrations when compared to FragPipe.

Although the imspy_dda pipeline yielded fewer peptide identifications, these results demonstrate the ease of implementing new functionalities to adapt a pipeline for challenging data sets. For those identified at 1% FDR, we show that there are high overlaps of identifications with other tools, which encourages further investigation into the initial separation capabilities in the future.

Rescoring had a high impact on identifications, more than doubling the number of found peptides in all cases. Indeed, it has been previously observed that rescoring is able to greatly increase the amount of identifications for immunopeptidomics-data.^13,20,22^

### Rescoring and Model Fine-Tuning

**Figure 5:**
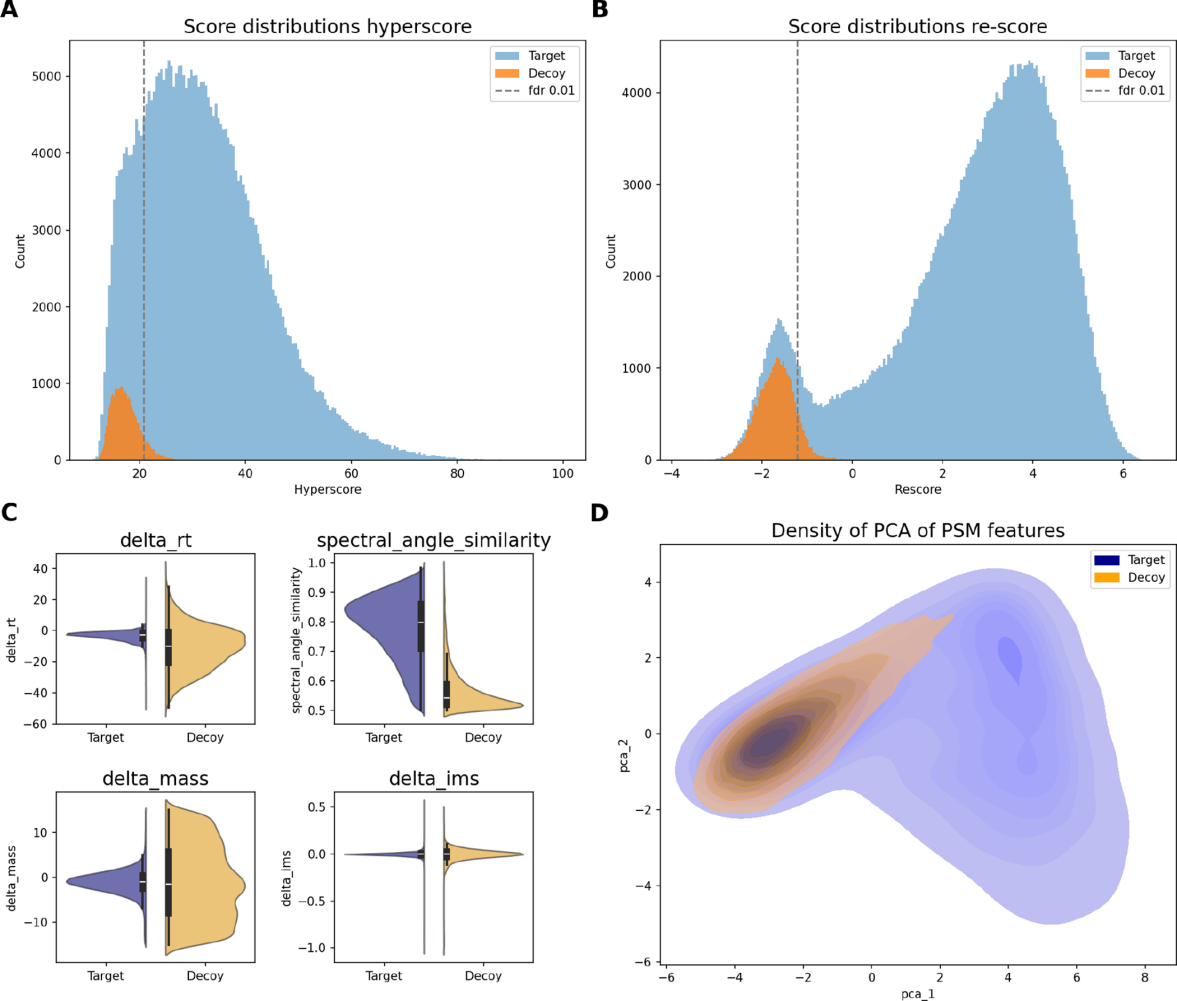
Visual insight into the process of rescoring on the HeLa-110 min data set. (A) Initial score distributions of target (blue) and decoy (orange) PSMs, generated using sagepy, which implements the X!Tandem score. (B) Score distributions after fitting a linear discriminant analysis (LDA) model, which maps PSMs from the feature space to new scores. PSMs are filtered to include only target and decoy hits of rank 1. See Figure S2 for the distribution of all PSM scores, independent of their rank. (C) Differences in the distributions of target and decoy hits for a collection of four selected features. Distributions are shown for all decoys, but target hits are filtered at 1% FDR. (D) Principal component analysis (PCA) illustrating the projection of the higher-dimensional feature space of target (blue) and decoy (orange) hits into two dimensions.

Rescoring has gained interest in computational proteomics^41^ as it routinely and significantly increases the number of identified peptides.^20,22^ The process is visually summarized in Figure 5, where the top panel shows how the score distributions are transformed from the initial scoring function (A) to the rescored scores (B), which enhances the differentiation of target and decoys. We point out that this task is actually a surrogate for the real objective to better separate true from false positive identifications from the target database. The lower panel depicts how the differences in a collection of features per peptide like retention time, spectral angle, or ion mobility between target and decoys provide information to find a better separation between them (C). A principal component projection of the full feature space into two dimensions is also shown (D), depicting the high-dimensional feature distribution in two dimensions. It can be observed that the density of target (blue) hits is bimodal, where one mode follows the unimodal distribution of the decoys (orange). This is expected, as it fits the underlying assumption that the distribution of decoys follows that of the false positives (left mode) and that those are partially separable from the true identifications (right mode).

Since the PSMs generated by sagepy are directly available within the pipeline environment, it was straightforward to use initial hits with high scores to fine-tune retention time and ion mobility predictors, similar to the already established fine-tuning of collision energies for intensity prediction.^13^ This also circumvents the typical postprocessing of retention time predictor outputs, where models trained on dimensionless indexed retention time values need to be adjusted to the experiment gradient lengths.^41^ In our experiments, fine-tuning of retention time and ion mobility did not significantly increase or decrease identifications. Nevertheless, this demonstrates the easiness of implementing such nodes into a pipeline using the *rustims* framework.

## Current Limitations

### TDF Support on macOS

The software is provided as prebuilt binaries for Linux, Windows, and MacOS, offering full functionality across all operating systems. However, reading raw data on macOS is limited due to the TDF reader’s dependency on Bruker binaries. While Windows and Linux users can fully access the raw data, MacOS users can only read indexed values, without access to Bruker’s proprietary conversion functions for translating TOF indices to *m*/*z* values and scans to inverse ion mobility values. Despite this, MacOS users can still utilize other features, such as Python-based interaction with Sage and analysis of MGF files.

### Performance on HLA-I Peptide Data

The ability of our framework to identify HLA-I peptides is currently lower than that of state-of-the-art software. However, the number of identified peptides can be significantly increased by utilizing the TIMS^2^Rescore rescoring tool, with which the outputs from our software are fully compatible (see Figure S5). Additionally, even with database chunking, significant computational resources are required. Nonetheless, to our knowledge there are currently no free and open-source solutions for processing HLA-I peptide data acquired on timsTOF platforms, making the availability of such a tool valuable for the community.

### Processing of DIA Data

Although the timsTOF platform is widely used for DIA acquisition, our current software does not yet support DIA data processing. In DIA, the cofragmentation of multiple precursors, rather than targeting high-intensity precursors with narrow selection windows, produces complex spectra composed of signals from multiple ions. As a result, most DIA raw data processing tools adopt peptide-centric approaches that rely on spectral libraries rather than in silico generated reference databases. While recent advancements have explored spectrum-centric approaches to DIA processing,^42,43^ adapting our framework for this type of data would require significant modifications. We plan to add DIA support in future releases.

## Conclusions

We present rustims, a collection of software tools for processing timsTOF raw data. rustims utilizes a two-language approach, where lower-level Rust code, specifically the rustdf and mscore crates, is exposed to Python via PyO3 bindings through the imspy-connector. Additionally, we developed bindings to the well-established Sage search engine via the sagepy-connector crate. The Python interfaces imspy and sagepy provide easy access, seamless integration into existing workflows, and enable fast prototyping, both being available on PyPi. We demonstrate the pipeline-building capabilities for processing raw DDA data acquired on a Bruker timsTOF by providing a command-line tool, the imspy_dda pipeline. We show the ability to parse, search, score, and rescore data by comparing results with FragPipe and PEAKS, achieving good identification rates and high overlap in peptide findings for tryptic samples. In addition, we exploited the flexibility of our framework to adapt the pipeline for immunopeptidomics data sets. The rustims project comprises a valuable addition to the free-and-open-source community, encouraging both experienced coders as well as newcomers to prototype and build solutions for processing of timsTOF acquired raw data.

## Data Availability

The mass spectrometry raw data sets have been deposited to the ProteomeXchange Consortium^44^ via the jPOSTrepo partner repository^45^ with the data set identifiers PXD043026 for ProteomeXchange and JPST002158 for jPOSTrepo for tryptic, HLA class I immunopeptidomics (HLA1p) with the data set identifiers PXD040385, JPST002044. All intermediate data generated during processing of the raw data sets and training data used for deep learning model training is available at Zenodo,^46^ together with jupyter notebooks to recreate all presented plots or retrain any of the used deep learning models.

## Supporting Information Available

Creation of a working imspy environment: step-by-step instructions for setting up an imspy Python environment; download of the results: guide to downloading data files from the Zenodo repository; downloading of RAW.d files and rerunning of the imspy_dda pipeline: instructions for downloading RAW data and executing the pipeline; HeLa samples: details on processing HeLa samples; HLA samples: details on processing HLA samples; recreating plots: scripts and Jupyter notebooks for plot reproduction; recreating trained machine learning models: scripts for training machine learning models used in the study; MGF creation with Compass DataAnalysis 6.1 (Bruker): instructions how to use the Compass DataAnalysis software to create MGF files from timsTOF DDA data; creation of a Docker image: instructions how to install the here presented collection of software packages and the full imspy_dda pipeline inside a Docker image; performance comparison of the imspy_dda pipeline with and without mokapot vs FragPipe + MSBooster on different gradient lengths of tryptic HeLa digest acquired with DDA-PASEF on the peptide level; distribution of hyperscores for target and decoy hits for all ranks; performance comparison of running Sage command line with Bruker vendor software extracted MGF files, Sage command line with TDF inputs, and imspy_dda; performance comparison of outputs from sagepy with TIMS^2^Rescore and imspy_dda with and without mokapot as postprocessor on the HeLa data sets; and comparison of outputs from PEAKS with MS^2^Rescore, sagepy with TIMS^2^Rescore, and imspy_dda on the HLA data sets (PDF)
