# Supplementary material for: Rustims: An Open-Source Framework for Rapid Development and Processing of timsTOF Data-Dependent Acquisition Data
Source: J Proteome Res. 2025 Apr 22;24(5):2358–68. doi: 10.1021/acs.jproteome.4c00966 (PMC12053931; doi:10.1021/acs.jproteome.4c00966)
Supplement: Supplementary file 1 — pr4c00966_si_001.pdf [file pr4c00966_si_001.pdf]

Supporting Information for: Rustims: An Open-Source Framework for Rapid Development and Processing of timsTOF Data-Dependent Acquisition Data

David Teschner,[*,†,‡] David Gomez-Zepeda,[¶,§] Mateusz K. Łącki,[‖] Thomas Kemmer,[†,‡] Anne Busch,[†,‡] Stefan Tenzer,[‖,¶,§] and Andreas Hildebrandt[*,†,‡]

†*Institute of Computer Science, Johannes-Gutenberg University, 55128 Mainz, Germany*

‡*Institute for Quantitative and Computer Biosciences (IQCB), Johannes-Gutenberg University, 55128 Mainz, Germany*

¶*Helmholtz Institute for Translational Oncology (HI-TRON) Mainz - a Helmholtz Institute of the DKFZ, Mainz, Germany*

§*German Cancer Research Center, DKFZ, 69120 Heidelberg, Germany*

‖*University Medical Center, Johannes-Gutenberg University, 55131 Mainz, Germany*

E-mail: dateschn@uni-mainz.de; andreas.hildebrandt@uni-mainz.de

Table of Contents

- Documentation
- 1. Creation of a Working `imspy` Environment
- 2. Download of the Results

- 3. Downloading of RAW `.d` Files and Re-running of the `imspy_dda` Pipeline
  - 3.1. HeLa Samples
  - 3.2. HLA Samples
  - 3.3. Recreating Plots
  - 3.4. Recreating Trained Machine Learning Models
- 4. MGF creation with Compass DataAnalysis 6.1 (Bruker)
- 5. Docker image
- Supporting Figures

Documentation

The two-language approach and the split into two repositories can make browsing the code base challenging at first. We provide an overview of how to easily navigate the main resources to understand the library. Users most likely want to start exploring the `imspy` and `sagepy` Python packages.

- **rustims project**: github.com/theGreatHerrLebert/rustims
- **sagepy project**: github.com/theGreatHerrLebert/sagepy
- **imspy example usage**: github.com/theGreatHerrLebert/rustims/tree/main/imspy
- **sagepy example notebooks**: github.com/theGreatHerrLebert/sagepy/tree/main/sagepy/examples
- **imspy_dda notebook**: github.com/theGreatHerrLebert/rustims/blob/main/imspy/examples/imspy_d[illegible]
- **imspy_dda implementation**: github.com/theGreatHerrLebert/rustims/blob/main/imspy/imspy/timst[illegible]
- **Documentation rust crate mscore**: thegreatherrlebert.github.io/rustims/main/mscore/

- **Documentation rust crate rustdf**: thegreatherrlebert.github.io/rustims/main/rustdf/
- **Documentation Python package imspy**: thegreatherrlebert.github.io/rustims/main/imspy/
- **Documentation Python package sagepy**: thegreatherrlebert.github.io/rustims/main/sagepy/
- **mscore on crates.io**: https://crates.io/crates/mscore/
- **rustdf on crates.io**: https://crates.io/crates/rustdf

1. Creation of a Working `imspy` Environment

We strongly recommend using a Linux-based environment, preferably Ubuntu $\geq$ 22.04, since TensorFlow does not have GPU support on Windows, which will slow down the machine learning parts of the pipeline considerably. Start by creating a virtual environment with Python 3.11.

```
# Create a virtual environment
python3.11 -m venv imspy

# Activate the virtual environment
source imspy/bin/activate

# Install imspy
pip install imspy

# (Optional) Install TensorFlow with GPU support
pip install tensorflow[and-cuda]==2.15.*
```

You can test if `imspy` has been successfully installed by running:

```
# Print out imspy_dda options
imspy_dda --help
```

This should print out the pipeline options. If it does, the tool has been installed successfully.

2. Download of the Results

Download the `rustims-submission.zip` file from this Zenodo repository and extract it to a location of your choice. After you have downloaded and extracted the folder, change into it:

```
cd rustims-submission/imspy_dda/
```

3. Downloading of RAW `.d` Files and Re-running of the `imspy_dda` Pipeline

3.1. HeLa Samples

The raw data used and their grouping according to gradient lengths can be found in the file `sample_groups.csv` available from this repository.

You can also find the reference proteome FASTA file and the `config.toml` file there. The respective raw data files can be found at ProteomeXchange with the identifier: PXD043026. For convenience, a bash script named `download.sh` for downloading and extracting the raw mass spectrometry TDF files is provided. For example, run:

```
./download.sh hela20
```

This will automatically download the 3 RAW `.d` files from ProteomeXchange and place them together into a subfolder. Next, run (this requires that the Python environment you installed `imspy` into is active):

```
imspy_dda hela/hela20/ hela/UP000005640.fasta \
--config hela/config_tryptic.toml
```

After the pipeline completes, results will be placed in: `hela/hela20/imspy/`, most importantly the files `PSMs.csv` and `Peptides.csv`.

3.2. HLA Samples

The raw data used and their grouping according to gradient lengths can be found in the file `sample_groups.csv` available from this repository.

You can also find the reference proteome FASTA file and the `config.toml` file there. The respective raw data files can be found at ProteomeXchange with the identifier: PXD040385. For convenience, a bash script named `download.sh` for downloading and extracting the raw mass spectrometry `.d` files is provided. For example, run:

```
./download.sh hla5
```

This will automatically download the 3 RAW `.d` files from ProteomeXchange and place them together into a subfolder. Next, run (this requires that the Python environment you installed `imspy` into is active):

```
imspy_dda hla/hla5/ hla/human_20365.fasta \
--config hla/config_hla.toml -fbs 6
```

Processing data with unspecific cleavage requires a lot of RAM. If the script crashes, you can try to make it work by increasing the number of splits of the FASTA file, e.g., `-fbs 50`. After the pipeline completes, results will be placed in: `hla/hla5/imspy/`, most importantly the files `PSMs.csv` and `Peptides.csv`.

3.3. Recreating Plots

The corresponding scripts, along with all intermediate results, are provided as Jupyter notebooks, e.g., `rustims-submission/imspy_dda/hela/HeLaImspyDDAFragpipeComparison.ipynb`.

3.4. Recreating Trained Machine Learning Models

The corresponding scripts, along with all used training data, are provided as Jupyter notebooks, e.g., `rustims-submission/re-scoring/models/ionmob/TrainingGruCCSPredictor.ipynb`.

4. MGF creation with Compass DataAnalysis 6.1 (Bruker)

1. open Compass DataAnalysis 6.1
2. open the .d files of interest. Multiple files can be opened together and MGF creation can be scheduled once producing MGFs independent of each other.
3. on the analysis list (at the left), select file or files that you want to process (multiple files can be selected). A file is selected if the entire field pertaining to it gets to be gray and stays gray.
4. On the task bar, click on Method > Default Application Methods > QTOF > Proteomics > Shotgun PASEF ProteinAnalysis.m
5. On the task bar, click on Method > Run.

This will schedule creation of mgf files. New mgf files will appear in the .d folders. During that operation, the cursor turns into a rotating blue circle and a progress bar appears (at least for part of calculation) for one .d folder after another. You can monitor the progress by seeing which of the selected folders are still selected in gray: once an MGF dump completes for a .d folder, it is simply deselected.

5. Docker image

For ease of use and reproducable running of our software, we are now providing a docker image for the AMD64 architecture. It can be also used on macOS ARM64 via virtualization. To get

the image, visit the mother-repository of the rustims project on GitHub and follow instructions provided in the README.md file.

Supporting Figures

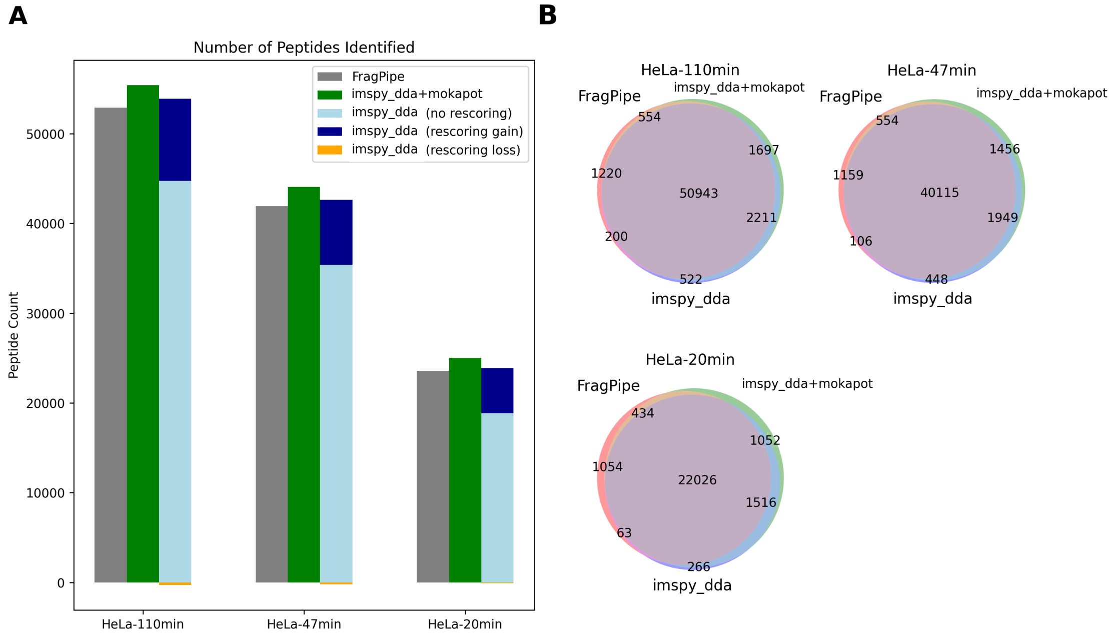

Figure 1: **Supporting Figure 1. Performance of `imspy_dda` pipeline with and without mokapot vs. FragPipe + MSBooster on different gradient lengths of tryptic HeLa digest acquired with DDA-PASEF on peptide level. A)** Total number of identified peptides at 1% FDR, showing that the identification pipeline presented here is able to achieve comparable performance to state-of-the-art software. **B–D)** Overlap of identified peptides at 1% FDR, showing high overlap for identifications from all tools and post-processing strategies.

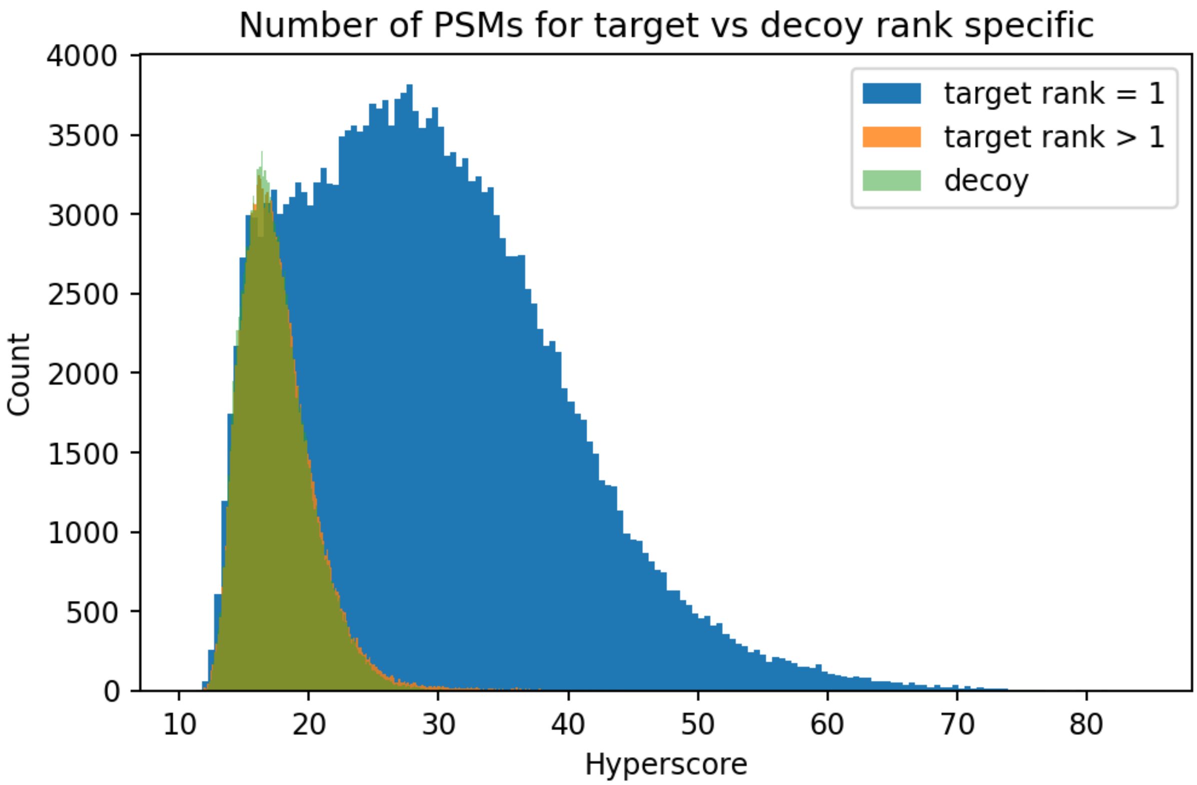

Figure 2: **Supporting Figure 2. Distribution of hyperscores for target and decoy hits for all ranks on dataset HeLa-110min.** Target hits with a rank higher than 1 follow the distribution of all decoy hits.

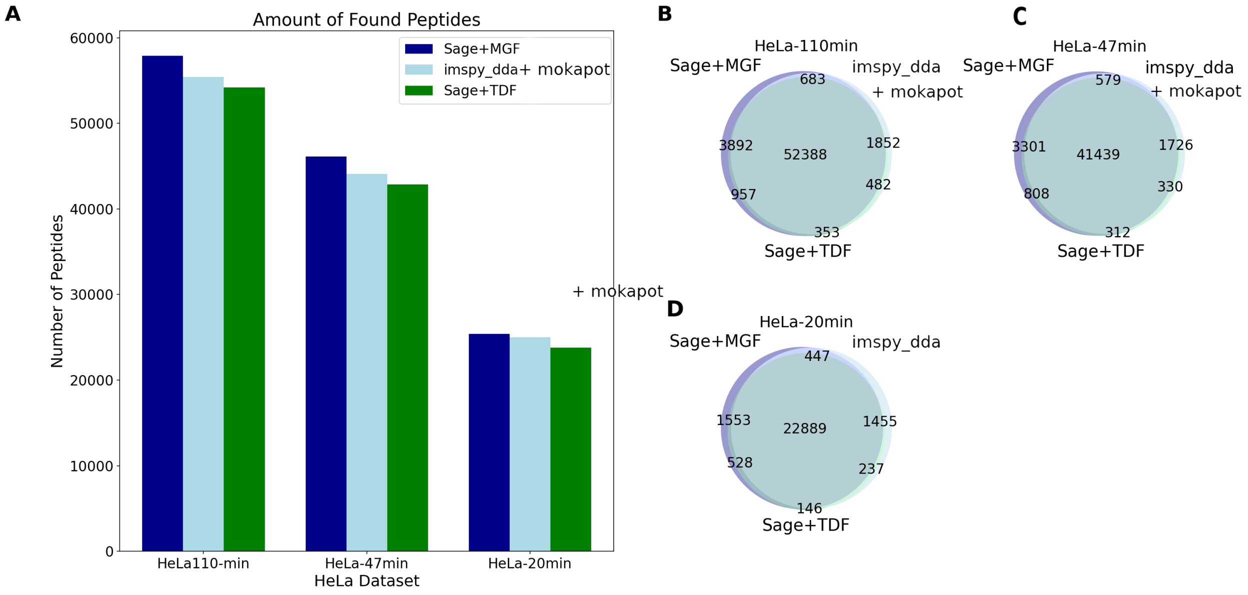

Figure 3: **Supporting Figure 3. Performance comparison of running the Sage command line tool with Bruker vendor software extracted MGF files, Sage command line with TDF inputs, and imspy_dda.** Using vendor software extracted MGF files yielded roughly 5% more significant peptides, indicating that spectral extraction and pre-processing can be improved in both our pipeline and the Sage command line tool.

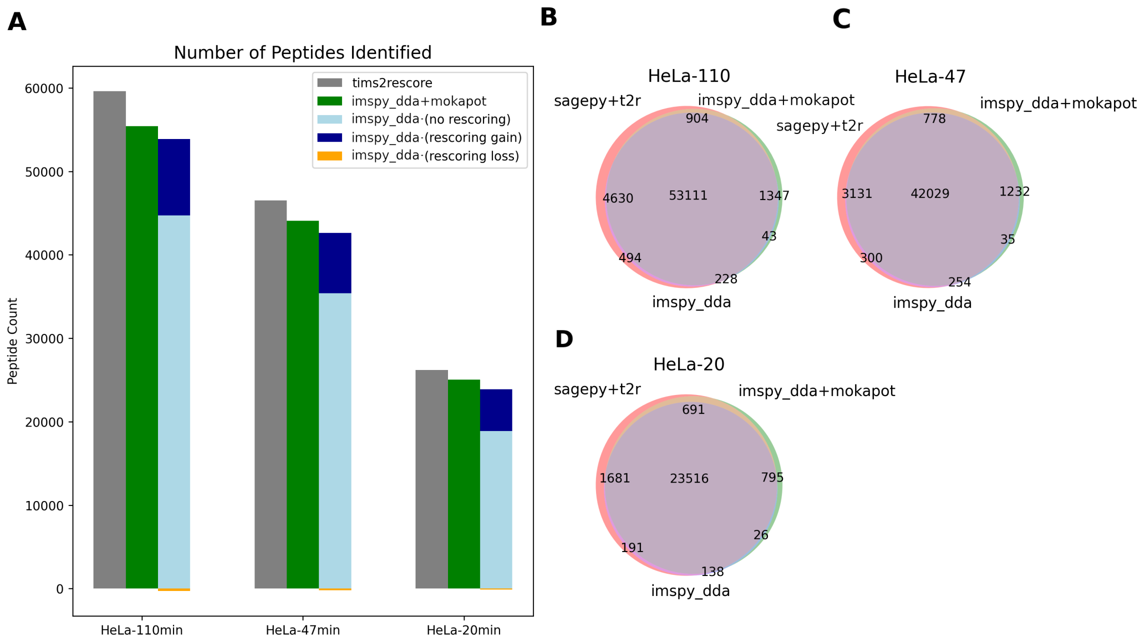

Figure 4: **Supporting Figure 4. Performance comparison of outputs from sagepy with tims2rescore and imspy_dda with and without mokapot as post-processor on the HeLa datasets.**

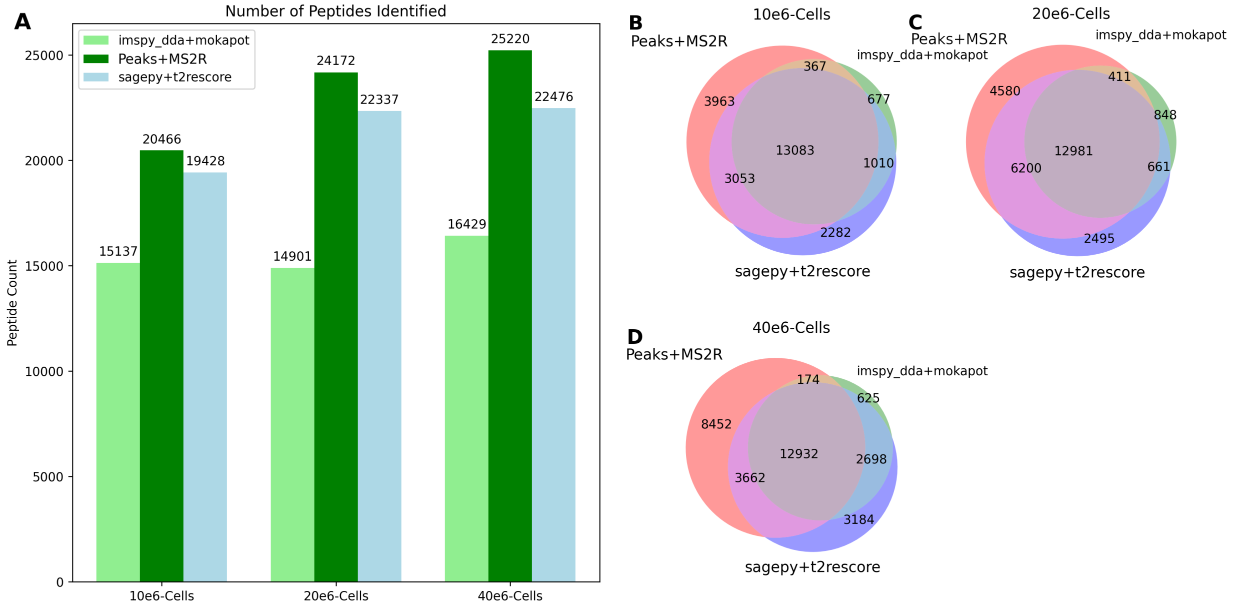

Figure 5: **Supporting Figure 5. Comparison of outputs from PEAKS with ms2rescore, sagepy with tims2rescore, and imspy_dda with mokapot on the HLA datasets.**
